# Chromatin modifications and the DNA damage response to ionizing radiation

**DOI:** 10.3389/fonc.2012.00214

**Published:** 2013-01-22

**Authors:** Rakesh Kumar, Nobuo Horikoshi, Mayank Singh, Arun Gupta, Hari S. Misra, Kevin Albuquerque, Clayton R. Hunt, Tej K. Pandita

**Affiliations:** ^1^Department of Radiation Oncology, University of Texas Southwestern Medical CenterDallas, TX, USA; ^2^Homi Bhabha National Institute, Molecular Genetics Section, Molecular Biology Division, Bhabha Atomic Research CentreMumbai, India

**Keywords:** histone modifications, DNA repair

## Abstract

In order to survive, cells have evolved highly effective repair mechanisms to deal with the potentially lethal DNA damage produced by exposure to endogenous as well as exogenous agents. Ionizing radiation exposure induces highly lethal DNA damage, especially DNA double-strand breaks (DSBs), that is sensed by the cellular machinery and then subsequently repaired by either of two different DSB repair mechanisms: (1) non-homologous end joining, which re-ligates the broken ends of the DNA and (2) homologous recombination, that employs an undamaged identical DNA sequence as a template, to maintain the fidelity of DNA repair. Repair of DSBs must occur within the natural context of the cellular DNA which, along with specific proteins, is organized to form chromatin, the overall structure of which can impede DNA damage site access by repair proteins. The chromatin complex is a dynamic structure and is known to change as required for ongoing cellular processes such as gene transcription or DNA replication. Similarly, during the process of DNA damage sensing and repair, chromatin needs to undergo several changes in order to facilitate accessibility of the repair machinery. Cells utilize several factors to modify the chromatin in order to locally open up the structure to reveal the underlying DNA sequence but post-translational modification of the histone components is one of the primary mechanisms. In this review, we will summarize chromatin modifications by the respective chromatin modifying factors that occur during the DNA damage response.

## INTRODUCTION

Cells derived from individuals with ataxia-telangiectasia are radiation sensitive and have a higher rate of conversion of DNA double-strand breaks (DSBs) into chromosome breaks post irradiation (Pandita and Hittelman, 1992a,b;Pandita and Richardson, 2009). The increased frequency of chromosome aberrations observed in ataxia-telangiectasia mutated (ATM) defective cells has been attributed to an altered chromatin status which is observed at both the global chromatin level as well as specifically at telomeric chromatin (Pandita and Hittelman, 1995;Smilenov et al., 1999;Pandita, 2001, 2002, 2003). It is now evident that DNA damage renders chromatin more sensitive to micrococal nuclease digestion (Telford and Stewart, 1989) and that induction of DSB results in decondensation of chromatin at the local as well as global level (Kruhlak et al., 2006;Dellaire et al., 2009), an essential requirement for activation of the DNA damage response (DDR) and subsequent DSB repair. The basic repeating unit of chromatin, the nucleosome, consists of a histone octamer complex containing four types of histones, namely H2A, H2B, H3, and H4 (Luger et al., 1997), around which is wound 146 bp of DNA. Nucleosome formation compacts naked double helical DNA by approximately six fold its linear dimension. DSB formation has been reported to lead to a localized disruption of nucleosomes, a process that depends on NBS1 and ATM (Berkovich et al., 2007). Histones consist structurally of a globular core domain with flexible tail regions at both the N-terminal and C-terminal regions. The globular core domain interacts with other histones while the termini contain highly conserved, charged lysine and arginine amino acid residues involved in DNA–histone interaction. In addition to histones, non-histone proteins are also involved in developing special chromatin structures. The known histone modifications induced following ionizing radiation exposure are phosphorylation, acetylation, methylation, and ubiquitination (Pandita and Richardson, 2009;Deem et al., 2012). Among these major chromatin modifications, post-damage-induced histone phosphorylation has been the most thoroughly studied. Recent studies have provided evidence that histone acetylation of lysine is also critical for the DDR. Post-translational modifications (PTMs) of histone proteins are critical for cellular recognition of DNA damage and subsequent recruitment of repair protein complexes to the damage sites. Moreover, evidence is emerging suggesting that pre-existing chromatin modifications also play an important role in the DDR (Sharma et al., 2010).

## HISTONE MODIFICATIONS IN DNA DSB REPAIR

Histone modifications provide support for critical repair proteins that act on the DNA damage site by helping in the recruitment of specific protein DNA repair factors and also play a role in sensing the initial DNA damage. Histone modifications have been detected on lysine as acetylation, methylation, or ubiquitination modification, on arginine as methylation and on serine/threonine as phosphorylation. Except for ubiquitination, all these modifications alter histone/DNA electrostatic interactions and ultimately change chromatin dynamics and function by altering access of the cellular factors involved in DDR.

## HISTONE PHOSPHORYLATION

Phosphorylation of all histones has been reported to affect transcription, DNA repair, apoptosis, and chromosome condensation during mitosis (Hanks et al., 1983; Van Hooser et al., 1998). H2A phosphorylation has been extensively studied in response to DNA damaging agents (Paull et al., 2000) and phosphorylation specifically of the H2A variant H2AX occurs immediately (within a few minutes) after exposure to ionizing radiation. Phosphorylation of H2AX (Rogakou et al., 1998), the product being referred to as γ-H2AX, has been shown to be an essential event in the response to DNA strand breaks and is thought to modify the higher order chromatin structure at the damage site. In yeast, H2A is phosphorylated at Ser129 by the Tel1 and Mec1 kinases in response to various DNA damaging agents (Kouzarides, 2000, 2007; Paull et al., 2000; Foster and Downs, 2005) while in humans ATM and ATR (human ortholog for yeast Tel1 and Mec1, respectively) phosphorylate the corresponding Ser139 of H2A. This phosphorylation event is critical for the recruitment of signaling/repair proteins to DNA damage sites since ionizing radiation induced formation of γ-H2AX foci is rapid, precedes repair factor assembly into repairosome foci and is required for subsequent foci formation by numerous factors including 53BP1, NBS1, BRCA1, and MDC1 (reviewed in Fernandez-Capetillo et al., 2004b). In addition, γ-H2AX has been shown to physically interact with NBS1, 53BP1, and MDC1 and deficiency of H2AX results in enhanced genomic instability, further supporting the role of γ-H2AX in DDR (reviewed in Fernandez-Capetillo et al., 2004b). Ionizing radiation (IR)-induced phosphorylation of H2AX occurs in all phases of the cell cycle, which is consistent with the fact that IR-induced ATM activation occurs in all phases of the cell cycle (Pandita et al., 2000). Phosphorylation of H2AX facilitates damage site recruitment of the DDR component MDC1 which binds to γ-H2AX via its BRCT domain (Yuan et al., 2010). γ-H2AX-recruited MDC1 is phosphorylated by ATM as well as by casein kinase 2 (Stucki et al., 2005; Lou et al., 2006). Binding of MDC1 to γ-H2AX is further modulated by males absent on the first (MOF)-dependent H4K16 acetylation (Li et al., 2010). Consistent with this, H2AX phosphorylation has been shown to facilitate the recruitment of SW1/SNF and RSC remodeling complexes and several repair proteins including RAD16, CSB/RAD26, RAD5, and RAD54 that belong to the SWI2/SNF2 family of helicases involved in DNA repair (Widlak et al., 2006). In addition to H2AX, other histones also undergo phosphorylation as part of the DDR, e.g., phosphorylation of H2B at Ser14 and N-terminal phosphorylation of H4 (Fernandez-Capetillo et al., 2004a). Phosphorylation of H2B and H4 has been reported to be abundant in close proximity to endonuclease-induced double-strand breaks (Cheung et al., 2005; Utley et al., 2005) and is mediated by sterile 20 kinase (Mst1; Ahn et al., 2005) and casein kinase 2 (Cheung et al., 2005; Utley et al., 2005). The major types of phosphorylated histones in chromatin and the associated kinases are summarized in **Table 1**.

**Table 1:** Phosphorylation.

Modification	Modifier	Histone	Role in DNA repair	Reference
pS139	ATM, ATR, DNA-PKcs	H2AX	Recruits and accumulates DDR proteins (MDC1) to the repair lesion and promotes histone acetylation	Rogakou et al. (1998), Kouzarides (2000, 2007), Paull et al. (2000), Foster and Downs (2005)
pT142	WSTF	H2AX	Recruits activated ATM and MDC1	Cook et al. (2009), Xiao et al. (2009)
pT14	CK2	H4	Promotes NHEJ by 53BP1 recruitment and methylation of H4K20	Fernandez-Capetillo et al. (2004a)
pS1	CK2	H4	Role in DDR	Cheung et al. (2005), Utley et al. (2005)

## HISTONE METHYLATION

Histone methylation on lysine and arginine (Grant, 2001; Zhang and Reinberg, 2001; Fischle et al., 2005; Kouzarides, 2007) was discovered 40 years ago (Murray, 1964). Specific methylation sites linked to the DDR are summarized in **Table 2**. Multiple methylations of H3 and H4 are reported (mono-, di-, and trimethyl groups per residue) at K4, K9, K27, K36, K79 and R2, R8, R17, R26 for H3 and K20 and R3 for H4. Histone methyl transferases use S-adenosyl-methionine as a methyl donor for the catalytic process. Lysine methylation is mainly commenced by the SET domain containing proteins [Dm Su(var)3-9, Enhancer of zeste (E(z)), and trithorax (trx)] while arginine methylation is carried out by co-activator arginine methyltransferase (CARM1) and the protein arginine methyl transferase (PRMT1). Additionally, as histone acetylation can be detected by bromodomain containing proteins, methylated sites are detected by proteins containing a chromodomain motif. Although dimethylation of histone H4 lysine 20 (H4K20me2), mediated by the histone methyltransferase MMSET (also known as NSD2 or WHSC1) in mammals (Pei et al., 2011), does not seem to increase globally after DNA damage, it is critical for 53BP1 recruitment to double-strand breaks (Pei et al., 2011) but does increase in the vicinity of DSBs. Not surprisingly then, MMSET depletion significantly decreases H4K20 methylation at DSBs and the subsequent accumulation of 53BP1. Recruitment of MMSET to DSBs requires the γ-H2AX–MDC1 pathway; specifically, the interaction between the MDC1 BRCT domain and phosphorylated Ser102 of MMSET (Pei et al., 2011). Based on such observations, it is possible that a pathway involving γ-H2AX–MDC1–MMSET regulates the induction of H4K20 methylation on histones around DSBs, which, in turn, facilitates 53BP1 recruitment.

**Table 2:** Methylation.

Modification	Modifier	Histone	Role in DNA repair	Reference
K4me3	SET1	H3	Stimulates V(D) J recombination via recombination activating gene (RAG) complex	Wang et al. (2009), Stanlie et al. (2010)
K9me3	Suv3-9H1/ Suv3-9H2	H3	Interacts with HP1β, phosphorylates damage-induced HP1β, and activates TIP60	Ayoub et al. (2008), Sun et al. (2009)
K36me2	Metnase/SETMAR	H3	Accumulates NBS1 and Ku to stimulate NHEJ	De Haro et al. (2010), Beck et al. (2011)
K79me3	DOT1	H3	Recruits RAD9 in budding yeast	Bostelman et al. (2007)
K20me2	Suv4-20H1/Suv4-20H2;MMSET	H4	Recruits DDR and repair proteins	Sanders et al. (2004), Schotta et al. (2004), Pei et al. (2011)

## HISTONE ACETYLATION

Histone acetylation neutralizes positively charged lysine residues, altering the chromatin fiber intra- and inter-nucleosomal interactions to facilitate decondensation and enhance access to nucleosomal DNA (Kouzarides, 2000; Anderson et al., 2001). **Table 3** summarizes the histone acetylation enzymes with known connections to the DDR and their specific histone substrates. Acetylation is a dynamic histone marker regulated by the balance between histone acetyltransferases (HATs), which transfer an acetyl moiety from acetyl-coenzyme A to the ε-amino group of lysine, and histone deacetylases (HDACs) which remove the acetyl-group from histones (Shahbazian and Grunstein, 2007). Histone modification occurs in both the nucleus and cytoplasm by Type-A and Type-B HATs, respectively. Type-A HATs are responsible for chromatin dynamics in the nucleus. There are multiple Type A HAT families including the GNAT superfamily, MYST family, p300/CBP, nuclear receptor co-activators, TAFII250 and TFIIIC. Individual histones can contain modifications that differentially regulate chromatin functions. While acetylation of H3 and H4 histones increases interactions with components of the transcriptional machinery containing bromodomains, methylation of lysine 9 on H3 (H3K9) allows heterochromatin protein 1 (HP1) binding via the chromodomain to the chromatin, thereby inhibiting binding of the transcription machinery to DNA. The acetylation at the N-terminuses of H3 and H4 and at H3K56 plays an important role in genomic stability, DNA replication, and in the binding of chromatin assembly factor (CF1)–PCNA complex (Chen and Tyler, 2008). Acetylation has been detected at four lysines (K5, K8, K12, and K16) in the N-terminal tail of the H4 histone. Acetylation at K16 in H4 was observed on the *Drosophila* male X chromosome (Turner et al., 1992) and correlated with gene dosage compensation. The modification has also been implicated in the control of chromatin structure responsible for interaction of other proteins (Shogren-Knaak et al., 2006). The acetyl-transferase responsible for histone H4 acetylation at K16 is MOF (Gupta et al., 2005, 2008; Smith et al., 2005; Sharma et al., 2010). Acetylation at H4 K16 (H4K16ac) has been implicated in the proper compaction of chromatin 30-nm fibers (Shogren-Knaak et al., 2006). More importantly, lack of MOF also influences ATM activation (Gupta et al., 2005) and results in delayed appearance of IR-induced γ-H2AX foci (Sharma et al., 2010). Consistent with the influence of histone H4 K16 acetylation on ATM activation, HDAC inhibitor treatment results in global ATM activation even in the absence of DNA damage (Bakkenist and Kastan, 2003).

**Table 3:** Histone acetylation.

Modification	Modifier	Histone	Role in DNA repair	Reference
K5ac	TIP60	H2AX	Helps in K119ub of H2AX and removal of H2AX from chromatin	Kusch et al. (2004), Ikura et al. (2007)
K36ac	CBP/p300	H2AX	Recruits Ku during NHEJ	Deem et al. (2012)
K9ac, K14ac, K23ac K27ac	GCN5,CBP/p300	H3	Recruits SW1/SNF complex, promotes spreading of γ-H2AX domain and regulates ATM activity	Tamburini and Tyler (2005)
K18ac	GCN5,CBP/p300	H3	Regulates Ku protein recruitment during DSB repair	Ogiwara et al. (2011)
K56ac	GCN5,CBP/p300, RTT109	H3	Depletes after IR to promote NHEJ, enriches K56ac after HR and UV repair	Miller et al. (2010)
K5ac, K8ac, K12ac, K16ac	MOF, TIP60-TRRAP; CBP/p300	H4	Recruits DDR and repair proteins and recruits SW1/SNF nucleosome remodeling complex	Gupta et al. (2005, 2008), Sharma et al. (2010)

## HISTONE UBIQUITINATION

Ubiquitination is a cellular process that conjugates a 76 amino acid protein, ubiquitin, to the lysine ε-amino group of specific proteins as a result of which it acts as a signaling molecule to regulate protein function and stability. Ubiquitination of H2A at K119 is associated with transcriptional repression via polycomb repressive complex (de Napoles et al., 2004; Fang et al., 2004) and the E3 ubiquitin-protein ligase RNF2 or RING2 responsible for this ubiquitination is stimulated by RING finger-domain containing proteins like BMI-1 and RINGIA (Cao et al., 2005). Interestingly, BMI-1, RINGIA, and RINGIB are involved in DSB-associated H2A ubiquitination (Cao and Yan, 2012). Ionizing radiation induces ubiquitination of nuclear H2A and H2AX histones. Monoubiquitinated H2A is enriched in the satellite regions of genome where as the ubiquitination of H2B is mostly in transcriptionally active genes. Histone H2A and H2AX can also be polyubiquitinated by ubiquitin ligase complex. During DNA repair at the break site, RNF8 and RNF168 catalyze formation of lysine 63 linked polyubiquitination chains on histones H2A and H2AX (Doil et al., 2009; Stewart et al., 2009; Campbell et al., 2012). RNF8 is rapidly recruited to the sites of DNA damage in an MDC1-dependent manner through its functional FHA domain and RNF8 is required to recruit other repair factors (Huen et al., 2007; Kolas et al., 2007; Mailand et al., 2007; Shi et al., 2008; Marteijn et al., 2009; Mok and Henderson, 2012). RNF8-catalyzed ubiquitin modification does not lead to protein degradation because the polyubiquitin synthesized in the RNF8/UBC13-mediated pathways is a lysine-63 linkage, rather than the lysine-48 canonical signal for protein degradation. Recent studies have revealed that K63Ub synthesis is regulated by the deubiquitinating enzyme, OTUB1 (OTU domain-containing ubiquitin aldehyde-binding protein 1; Wiener et al., 2012). It is thought that RNF8 executed ubiquitination has a role in maintaining genomic integrity, however, the role of post-damage monoubiquitylation in chromatin reassembly needs to be elucidated (Deem et al., 2012). It has been reported that RNF8 is inactive toward nucleosomal histone H2A. In contrast RNF168 catalyzes the monoubiquitination of the histones (H2A/H2AX) specifically on K13-15 (Mattiroli et al., 2012). Interestingly, E3 ligases like BRCA1 promote BRCA2 recruitment that appears in turn to promote the recruitment of RAD51 involved in homologous recombination (HR; Qing et al., 2011). The major histone sites of ubiquitination, the enzymes required and the role of the modification in the DDR are summarized in **Table 4**.

**Table 4:** Ubiquitination.

Modification	Modifier	Histone	Role in DNA repair	Reference
K119ub/K119ub2, K119poly-ub	RNF8, RNF168	H2A	Accumulates BRAC1 and 53BP1 for DNA repair	Ikura et al. (2007)
K119ub/K119ub2, K119poly-ub	RNF8, RNF168, TIP60–UBC13	H2AX	Recruits DDR proteins to the repair lesion	Mailand et al. (2007), Doil et al. (2009), Stewart et al. (2009)
K120ub	RNF20–RNF40	H2B	Recruits XRCC4 and Ku for NHEJ and BRCA1 and RAD51 FOR HR	Moyal et al. (2011), Nakamura et al. (2011)
K91ub	BBAP	H4	Induces H4K20me modification and recruits 53BP1 to promote NHEJ	Yan et al. (2009)

## CHROMATIN MODIFICATIONS DURING NON-HOMOLOGOUS END JOINING REPAIR

A majority of the genes responsible for DSB repair in human cells have been identified and have been assigned to one of several different repair pathways. DNA DSBs induced by IR are mostly repaired by either non-homologous end joining (NHEJ) or HR (Cartwright et al., 1998; Jeggo, 1998; Haber, 2000; Rothkamm et al., 2003; Scott and Pandita, 2006; Sonoda et al., 2006; Pandita and Richardson, 2009; Chapman et al., 2012). For DNA DSB repair by NHEJ, a very little or no DNA sequence homology at the damaged ends is required. NHEJ in mammalian cells is thought to be the major pathway for DNA damage repair after ionizing radiation exposure. There are two types of NHEJ repair: (1) Canonical end joining pathway (C-NHEJ) and the (2) Alternative NHEJ (A-NHEJ) pathway, the later being a minor DSB repair pathway that works in specific situations such as the absence of Ku. C-NHEJ is essential for both somatic cell survival after IR treatment and V(D)J recombination, which generates the antibody and T cell receptor diversity required for lymphocyte maturation (Boboila et al., 2012). Binding of the ring shaped Ku70/Ku80 heterodimer (Ku) to both ends of a DSB initiates C-NHEJ at damaged DNA sites followed by loading of DNA-dependent protein kinase catalytic subunit (DNA-PKcs) onto the DNA-Ku complexes. Prior to loading of the repair proteins, histone modifications occur which facilitate the recruitment of repair proteins at the damage site. DNA-bound Ku proteins serve as a template for loading DNA-PKcs which help translocate into the duplex by one helical turn, leaving DNA-PKcs near the DNA terminus to assist in tethering the broken ends together and prevent exonucleolytic degradation of the DNA ends (Gapud and Sleckman, 2011). This also helps DNA strands search for micro homologies. Activated DNA-PKcs phosphorylates different components of the NHEJ machinery to facilitate end-processing; DNA-PKcs itself undergoes autophosphorylation, which helps release DNA-PKcs from the break site so that subsequent steps of C-NHEJ can occur. DNA-PK holoenzyme (Ku/DNA-PKcs) recognizes, protects and bridges the DNA ends. DNA-PK conformational autophosphorylation is necessary for activation of end-processing enzymes, such as the Artemis nuclease. This complex serves as a docking platform for DNA polymerases and ligation factors like DNA ligase IV and X-ray cross complementing factor 4 (XRCC4), which help in DNA joining. Cell deficient for the canonical NHEJ proteins undergo DSB repair by an alternative form of NHEJ (Alt NHEJ), which operates at telomeres in telomerase deficient cells or in the absence of Ku or DNA-PKcs. This pathway involves proteins different from those involved in the C-NHEJ pathway including poly(ADP-ribose) polymerase 1 (PARP-1), XRCC1, DNA ligase III (LIG3), polynucleotide kinase, or Flap endonuclease 1. Though this pathway is relatively poorly characterized, certain features of A-NHEJ are well characterized such as a slower kinetics of DSB repair than in C-NHEJ and competitive repression of A-NHEJ by Ku under normal conditions. Whether C-NHEJ and Alt-NHEJ require any pathway specific chromatin modifications is not yet known.

The first visible chromatin modification that occurs immediately after cellular exposure to IR is phosphorylation of histone H2AX on Ser139, the product being termed γ-H2AX, which functions to recruit repair proteins. Besides γ-H2AX, other modifications involved in the NHEJ are acetylation, methylation, phosphorylation, and ubiquitination of histone H2A, H2B, H3, and H4. These histone modifications may play a role in either marking the site of a DNA break to facilitate the recruitment of DNA repair proteins or relaxing the chromatin so the repair proteins can access the damaged DNA site. For example, Metnase, a methylase which dimethylates histone H3 residue K36 (H3K36me2) in the DNA DSB region, and the levels of H3K36me2 have been found to correspond positively to DSB repair efficiency (reviewed in Williamson et al., 2012). Another modification, H3K4me3, carried out by Set1p methyltransferase has been found at DSBs and the absence of this modification has been linked to poor DNA DSB repair (Faucher and Wellinger, 2010).

During NHEJ repair, histones H3 and H4 are reportedly acetylated on the N-terminal lysines (reviewed in Williamson et al., 2012). NuA4–TIP60 acetylates histone H4 at DSBs and this acetylation has been linked with improved DSB repair (Bird et al., 2002; Murr et al., 2006). Similarly, the base levels of H4K16ac prior to DNA DSB induction influences repair as cells with decreased levels of H4K16ac have reduced efficiency of DNA DSB repair by NHEJ (Sharma et al., 2010). Other histone modifications by INO80, SWR1, and RSC complexes are necessary for recruitment of Ku70/80 at DNA DSB sites (Shim et al., 2007; van Attikum et al., 2007). It is interesting to note that at DSBs, RSC complex recruits Mre11, which is followed by ATPase remodeling to facilitate access to the site by proteins required for NHEJ-mediated repair (Shim et al., 2007). Furthermore, acetylation of H3 and H4 by CBP and p300 cooperate with the SW1/SNF complex to facilitate recruitment of Ku70/80 (Ogiwara et al., 2011). The function of histone acetylation therefore seems to be during the early stages of NHEJ to facilitate chromatin relaxation and subsequently allow the repair proteins to access the DNA DSB.

## CHROMATIN MODIFICATIONS DURING HOMOLOGOUS RECOMBINATION REPAIR

During the DNA DSB repair by HR, nucleotide sequences are exchanged between two similar or identical molecules of DNA, allowing the cells to accurately repair DNA. HR type repair is predominant during meiosis and in the S- and G2-phases of the cell cycle, whereas NHEJ repair is predominant in G1-phase cells. HR repair is a complex process, which requires significant alterations in chromatin structure in order to allow repair proteins access to damaged DNA. During this process, the first step is the processing of the exposed DNA ends to produce 3′-overhanging DNA ends for RAD51 binding. Resection is followed by RAD51 filament formation on the resulting single-stranded DNA, homologous sequence search, heteroduplex formation, repair synthesis, and resolution of the heteroduplex, all steps that require major changes in the chromatin structure. While HR makes a significant contribution to cell survival after IR exposure only in the S- and G2-phases, replication-associated one-ended DSBs are also efficiently and primarily repaired by HR.

The initiating step for HR is end-resection to create a 3′-overhang, which is subsequently coated by replication protein A (RPA). After formation of a DSB end, these ends are resected in vertebrates by the MRE11/RAD50/NBS1(MRN) complex or the MRX complex in yeast. Exo1 along with DNA2 nuclease then digest the 5′-end in eukaryotes. In yeast, the MRX complex, in cooperation with Sae2, is important for initiating DNA resection. The MRN complex serve as a multipurpose DNA clamp that acts to directly bridge severed DNA ends. DNA resection is followed by a critical step, which is recognition of homologous DNA sequences followed by generation of a joint molecule between single-stranded DNA and its duplex repair template. DNA strand exchange leads to switching of base-paired partners between these DNAs. Homology recognition and strand exchange are mediated by recombinase proteins such as RecA in prokaryotes and RAD51 in eukaryotes. RecA and RAD51 assembled into protein filaments on single-stranded DNA catalyze DNA rearrangement aided by accessory/mediator proteins. Subsequent steps in HR can include the engagement of the second DNA end, DNA branch migration, and eventual resolution of repaired DNA strands.

Upon joint molecule formation, a critical common step in the HR pathways is the hand off of the 3′-end of the incoming DNA molecule to a DNA polymerase, such that recombination will result in two restored duplex DNAs. At this stage of the reaction the partner DNA molecules are physically joined via branched DNA structures, which upon ligation can be converted into so-called Holliday junctions. There appear to be many structure-specific endonucleases that can resolve these structures resulting in the completion of DNA repair by HR. Enzymes such as the *Escherichia coli* RuvABC complex and the eukaryotic Gen1 protein incise Holliday junctions producing directly ligatable crossover and non-crossover products. Alternatively, a DNA helicase, BLM, in combination with a type I topoisomerase, can resolve Holliday junctions. Branched DNA intermediates in HR can also be acted upon by evolutionary-conserved structure-specific endonucleases, including Mus81/Eme1 and Slx1/Slx4. During the HR process, a number of acetylation events occur on histones H3 and H4 with the proteins implicated in the modification being GCN5, NuA4, and HAT1 (Tamburini and Tyler, 2005; Murr et al., 2006). GCN5 also plays some role in pathway choice for DSB repair as DNA-PKcs phosphorylates GCN5 to inactivate its HAT domain. In addition, GCN5 also interacts with BRCA1 through a mechanism that is dependent upon its HAT activity, suggesting a role in HR repair of DNA DSBs (Oishi et al., 2006).

During HR repair, MDC1 recruits RNF8 (E3) to ubiquitinate H2AX (Kolas et al., 2007; Mohammad and Yaffe, 2009). Subsequently, recruited BRCA1 further maintains H2AX ubiquitination in order to recruit downstream DDR and DSB repair components. Furthermore, at the completion of HR repair chromatin structure must be restored to the pre-damage state, a process that starts with dephosphorylation of γ-H2AX by protein phosphatase 2A (PP2A) or PP4C in mammals (Hanks et al., 1983). Removal of acetylation marks occurs by HDACs, which subsequently results in the condensation of chromatin back to its native configuration. 

Besides the chromatin modifying factors, a non-histone chromatin protein known as HP1, initially identified in *Drosophila* and named for its predominant localization to pericentric heterochromatin (Li and Smerdon, 2002), plays a role in genomic stability (Sharma et al., 2003). There are three mammalian HP1 isoforms termed HP1α, HP1β (M31) (Cbx1), and HP1γ (M32) (Singh et al., 1991; Eissenberg and Elgin, 2000; Jones et al., 2000). HP1β is a dosage-dependent modifier of pericentric heterochromatin-induced silencing (Festenstein et al., 1999). HP1β plays a critical role in maintaining genomic stability in mammalian cells (Sharma et al., 2003; Aucott et al., 2008) with both negative (Ayoub et al., 2008; Goodarzi et al., 2008) as well as positive (Luijsterburg et al., 2009) effects on DNA damage repair. First, damage-dependent phosphorylation of HP1β decreases its chromodomain-dependent affinity for H3K9me3 leading to transient displacement of HP1β from DNA damage sites (Ayoub et al., 2008). In addition, HP1β depletion alleviates the ATM requirement for efficient DSB repair in heterochromatic regions (Goodarzi et al., 2008), suggesting HP1β suppresses repair in heterochromatic DNA regions. However, HP1β was also found to accumulate at DNA damage sites, indicating a more active involvement in DNA repair (Luijsterburg et al., 2009). In human cells, HP1β overexpression increased IR-induced chromosomal damage (Sharma et al., 2003). HP1β mobilization during DNA repair is regulated by ATM-dependent KAP-1 Ser473 phosphorylation (Ziv et al., 2006; Bolderson et al., 2012).

## CONCLUSION AND FUTURE DIRECTIONS

While the mechanisms by which cells activate the DDR and repair DNA damage are becoming clear, much less is known about how the cell restores the pre-damage original chromatin structure once damage is repaired. In addition, there is growing evidence that besides post DNA damage alteration of histone modifications, pre-existing histone PTMs play a critical role in the DDR and subsequent repair. Histone modification patterns change during the induction of DNA DSB as well as during the repair process. These changing modifications allow the chromatin to open at the sites of DNA damage but some modifications can hold the chromatin in a condensed state. Pre-existing chromatin modifications at the histone level can directly affect the initial DDR and subsequent pathway choice for NHEJ and HR. Modifications like H4K16ac or H4K20me2,regulated by MOF and MMSET, respectively, are epigenetic markers that could act as a histone code since the absence of appropriate markers results both in the loss of a DDR and subsequent repair (Gupta et al., 2005, 2008; Sharma et al., 2010; Hajdu et al., 2011; Bhadra et al., 2012). It will be important to establish the relative impact of epigenetic changes in the context of DNA sequence and their role in DNA DSB repair. Modulating histone acetylation with HDAC inhibitors can alter the IR-induced response resulting in radioprotection as well as radiosensitization (Camphausen and Tofilon, 2007). Small molecule inhibitors of histone modifying enzymes, therefore, represent a promising means for modulating radiotherapy response. As with other areas of basic research, understanding the significance of chromatin modifications as histone code will help to tailor radiotherapy based on patient and tumor epigenetic phenotype.

## Conflict of Interest Statement

The authors declare that the research was conducted in the absence of any commercial or financial relationships that could be construed as a potential conflict of interest.
